# Sub-nanosecond light-pulse generation with waveguide-coupled carbon nanotube transducers

**DOI:** 10.3762/bjnano.8.5

**Published:** 2017-01-05

**Authors:** Felix Pyatkov, Svetlana Khasminskaya, Vadim Kovalyuk, Frank Hennrich, Manfred M Kappes, Gregory N Goltsman, Wolfram H P Pernice, Ralph Krupke

**Affiliations:** 1 Institute of Nanotechnology, Karlsruhe Institute of Technology, Karlsruhe 76021, Germany; 2 Department of Materials and Earth Sciences, Technische Universität Darmstadt, Darmstadt 64287, Germany; 3 Department of Physics, Moscow State Pedagogical University, Moscow 119992, Russia; 4 Institute of Physical Chemistry, Karlsruhe Institute of Technology, Karlsruhe 76021, Germany; 5 National Research University Higher School of Economics, Moscow 101000, Russia; 6 Institute of Physics, University of Münster, Münster 48149, Germany

**Keywords:** carbon nanotubes, infrared, integrated optics devices, nanomaterials

## Abstract

Carbon nanotubes (CNTs) have recently been integrated into optical waveguides and operated as electrically-driven light emitters under constant electrical bias. Such devices are of interest for the conversion of fast electrical signals into optical ones within a nanophotonic circuit. Here, we demonstrate that waveguide-integrated single-walled CNTs are promising high-speed transducers for light-pulse generation in the gigahertz range. Using a scalable fabrication approach we realize hybrid CNT-based nanophotonic devices, which generate optical pulse trains in the range from 200 kHz to 2 GHz with decay times below 80 ps. Our results illustrate the potential of CNTs for hybrid optoelectronic systems and nanoscale on-chip light sources.

## Introduction

Efficient transducers that allow converting electrical signals into optical ones and vice versa are essential ingredients for emerging applications in on-chip optoelectronic circuits. In particular, nanoscale transducers that can be seamlessly integrated into optical waveguide structures are needed to enable on-chip data communication in devices with small footprint. In this context carbon nanotubes (CNTs) have been identified as promising active components [1–2]. As a first step towards CNT-based optoelectronic photonic devices, light generation in waveguide-like electrodes [3] and optical waveguides [4–6] via electrically driven CNTs has been demonstrated. Very recently electroluminescent CNTs integrated into photonic circuits emerged as sources of non-classical light [7]. Besides electrical drive, optically stimulated light emission from CNTs coupled into waveguides [8] and to cavities [9] has been achieved. It was demonstrated, that the wavelength and the line shape of a CNT emission can be tailored by the photonic environment [6,10]. Beyond continuous wave generation of light, an important aspect that needs to be addressed is the question of how fast a waveguide-coupled CNT transducer can respond to an electrical signal. Indeed, time-dependent incandescence from a CNT film in free-space has been measured [11] and modulation of CNT emission with decay times below 250 ps has been shown [6]. The intrinsic characteristic timescale for CNT incandescence is expected to be of the order of 10 ps, estimated from the heat capacitance of the CNTs and the thermal coupling to the dielectric substrate and metallic leads [11]. Hence, CNT-based transducers operating at 100 GHz seem to be possible.

In this work, we investigated the dynamic response and coupling efficiency of waveguide-coupled CNT transducers to electrical signals and analyze the optical pulses propagating in the waveguide.

## Results and Discussion

### Fabrication of waveguide-integrated CNT emitters

**Figure 1:**
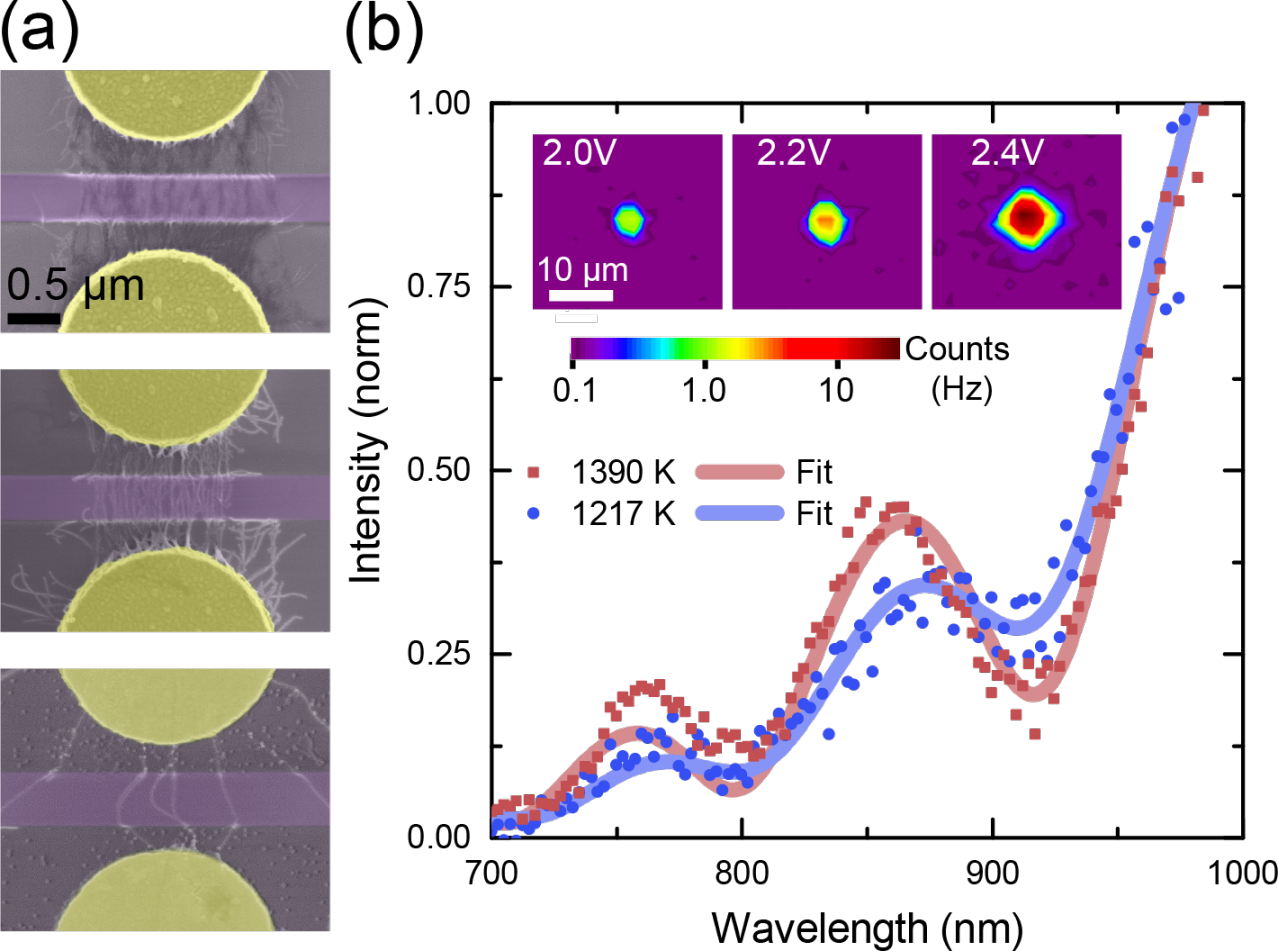
(a) Waveguide-integrated CNT transducers. False-colored scanning electron image of the waveguides (purple), horizontally aligned between metallic contacts (yellow). The CNTs can be seen as thin vertical lines between the electrodes crossing the waveguides. The density of CNTs varies from ca. 100 µm^−1^ to ca. 10 µm^−1^ to ca. 3 µm^−1^ (SEM images from the top to the bottom). (b) Emission spectra from a waveguide-integrated CNT transducer, driven with 200 ns electrical pulses at 200 kHz repetition rate for 2.5 V_DC_ + 2 V_pulse_ (blue circles) and 2.5 V_DC_ + 3 V_pulse_ (red squares). The full lines are fits to a black-body radiation curve modulated by a substrate-induced interference. The thermal emitter temperature from the fit is 1217 K (1390 K). Inset: spatially resolved light emission from a CNT emitter driven with 100 ns electrical pulses at 200 kHz rate for *V*_pulse_ = 2.0, 2.2, 2.4 V.

Our waveguide-coupled CNT (WG-CNT) transducers consist of three components: a rib waveguide, metallic contacts next to the waveguide, and metallic single-walled carbon nanotubes (SWCNTs) placed on top of the waveguide, bridging the contacts (Figure 1a). We use the design and fabrication approach for our samples that has been described in detail elsewhere [4]. Both electrodes and waveguides were defined using several steps of electron beam lithography on top of Si_3_N_4_/SiO_2_/Si substrate. Au/Cr contacts were produced by physical vapor deposition, and 600 nm wide, half-etched Si_3_N_4_-waveguides were formed with reactive ion etching. A typical sample contains tens of contact pairs and CNTs that were placed in between using dielectrophoresis (DEP) [12–13]. DEP allows for site-selective placement of CNTs onto electrodes with their long axis aligned perpendicular to the waveguide [4]. We used gel-filtrated metallic CNTs obtained from HiPco material [14]. The deposition density has been varied in the range of 1–100 CNTs per 1 µm waveguide length. After DEP the samples were baked for 1.5 h at 150 °C in ambient air in an oven to improve the contact adhesion. Several waveguide coupled CNT devices are shown in Figure 1a.

By connecting the driving electrodes of the CNT to a modulated voltage source, the nanoscale emitter can be driven into incandescence. We employ a pulse generator (Agilent 8131A for long pulses and Agilent 8133A for short electrical pulses) in combination with a Keithley 6430 source-meter for additional DC-biasing via a bias tee (ZFBT-6GHz, Mini-Circuits) to apply a time-varying voltage to the driving electrodes. Light emission from WG-CNT transducers was characterized with both a free-space setup and a fiber-coupled system. The free-space setup allows for the spatial and spectral analysis of light emitted from a transducer along the surface normal. The fiber-coupled system is dedicated to analyzing short light pulses, emitted by the transducer, and propagating within the waveguide.

### Light emission from waveguide-coupled CNTs under pulsed excitation

For analysis with the free-space setup, the transducers were placed in a lighttight system comprising of a Zeiss AxioTech Vario microscope, directly attached to an Acton SpectraPro 2360 spectrometer. The latter consists of a switchable mirror and grating, and is equipped with a PIXIS 256E CCD camera (Princeton Instruments) [15]. The spectrum of the light, emitted at the position of the CNT, is shown in Figure 1b, while the spatial distribution of the emission is presented in the inset. The signal intensity increases towards longer wavelengths with a superimposed intensity modulation. This behavior is characteristic for an incandescent emitter on a layered substrate that leads to interference fringes in the spectral profile, as observed for light emitting CNTs [4] and graphene [16]. The emitter temperature was extracted by fitting the data to a Planck spectrum


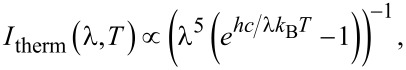


modulated by the substrate-induced interference [3]. Depending on the density of the CNTs as well as the biasing conditions, the temperature of the emitter lies within the range of 1000 to 1500 K, and increases with the pulse amplitude. The temperature of the CNT in the pulsed regime is similar to temperatures reached under DC-biasing [4,17].

**Figure 2:**
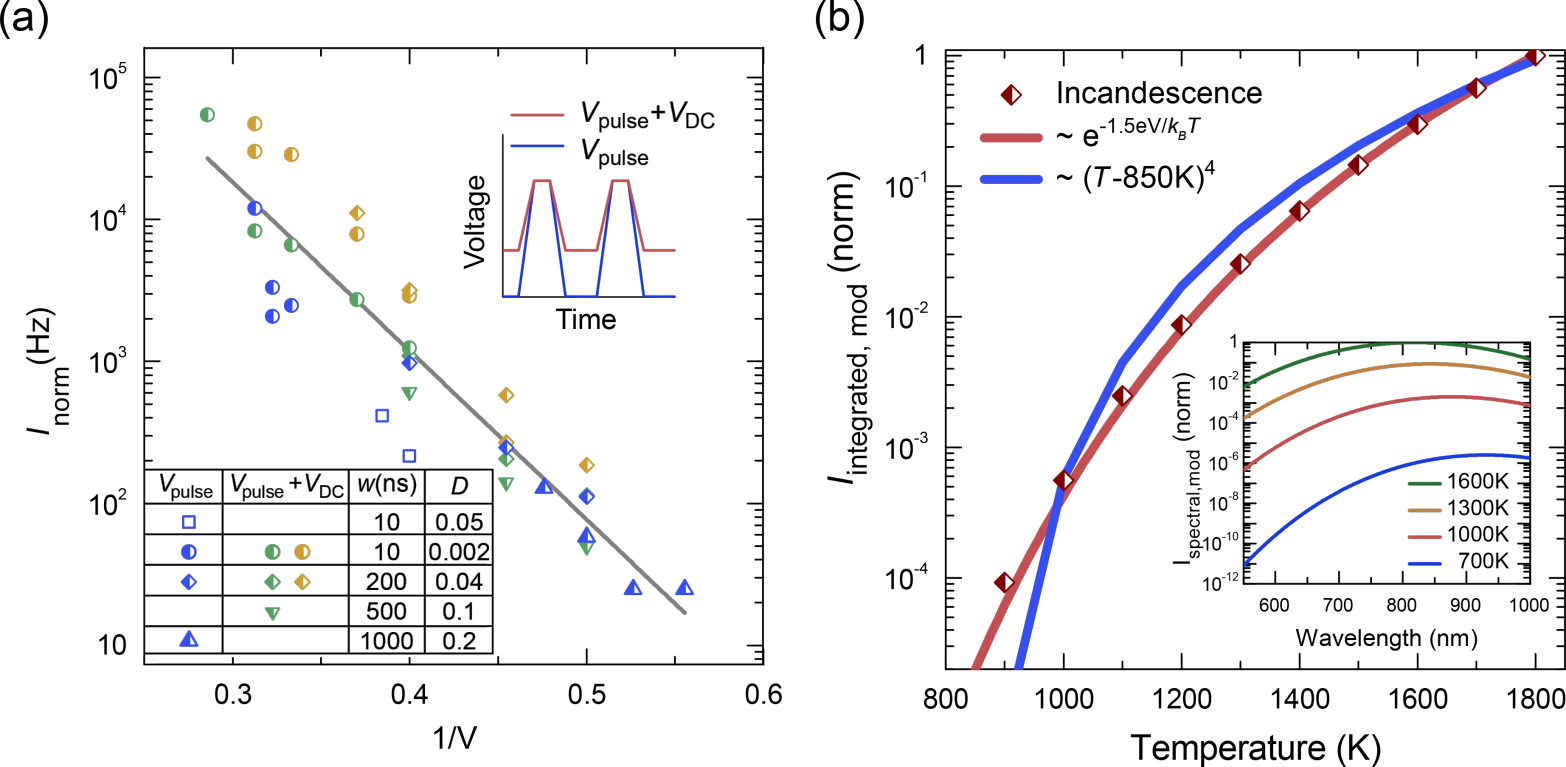
(a) Normalized total intensity *I*_norm_ versus 1/*V* under variation of electrical pulse width, *w*, and duty cycle, *D*. The full line is a fit to the data. Data acquired with an additional offset voltage *V*_DC_ = 1.5 V is labeled with green and yellow symbols, data acquired without offset is shown as blue symbols. The inset schematically illustrates two biasing schemes. All measurements refer to light propagating along the surface normal collected with the free-space setup. (b) Simulation of the temperature-dependent total intensity of the incandescent light collected with a CCD camera *I*_integrated,mod_(*T*) (red symbols), compared with exponential (red line) and power law (blue line) fits. Inset: Simulated spectra *I*_spectral,mod_(λ,*T*) of incandescent light sources emitting at the indicated temperatures. The shape of the spectra deviates from the Planck curve *I*_therm_(λ,*T*) because of the spectral sensitivity of the detector *S*(λ).

For a comprehensive characterization of the emitter response to a modulated electrical signal, we varied the pulse amplitude *V*_pulse_, the pulse width *w*, pulse period *T*, and thereby the duty cycle *D* = *w*/*T*. We measured the total intensity of the incandescent CNT emitters, *I*_E_*,* for each parameter set. Emission is observed above a device-dependent threshold *V*_th_ of about 1.5 *V*, which is independent of *w* and *D* over a broad frequency range, as shown in Figure 2a for contacts with similar CNT-density. The maximum RF amplitude of the pulse generator was not always sufficiently high to record light emission. Therefore a device-specific DC offset *V*_DC_ ≤ *V*_th_ was applied to raise the emission count rate slightly below the dark count rate of the system without RF contribution (Figure 2a, green and yellow symbols).

The emission was remarkably stable over hours. As expected, the absolute intensity depends on the duty cycle. Therefore, to consolidate all data sets into one graph, we have plotted in Figure 2a the normalized intensity *I*_norm_ = *I*_E_/*D* for pulses with 10 to 1000 ns width and duty cycles ranging from 0.002 to 0.2. As a general trend we observe that *I*_norm_ increases with *V* = *V*_pulse_ + *V*_DC_ by four orders of magnitude. Moreover, by plotting *I*_norm_ on a logarithmic scale against the inverse voltage *V*^−1^, the data collapses onto a straight line. This establishes the dependence 
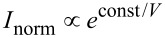
. For further understanding it is important to realize that the emission has been recorded in the high-bias regime where the current through the CNT reaches saturation, and the dissipated power and, hence, the temperature increases linearly with voltage [18–20]. As a result 
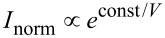
 can be converted into 
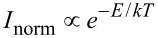
, similar to previous measurements with metallic CNTs [17]. To rationalize this dependence we have modeled the temperature-dependent spectral distribution *I*_spectral,mod_(λ,*T*) as the product of the Planck spectrum *I*_therm_(λ,*T*) and the spectral sensitivity of the detector *S*(λ) and plotted the result in the inset of Figure 2b. For simplicity, we have approximated the sensitivity of our detector by a Gaussian spectral profile centered at 830 nm and a FWHM of about 250 nm. From these spectra we calculated the wavelength-integrated, temperature-dependent intensity of the collected light *I*_integrated,mod_(*T*) presented in Figure 2b (red symbols). Since the limited detection range of the CCD camera allows for the detection of broadband CNT-emitted light only for *hc*/λ >> *kT*, the temperature dependence of *I*_integrated,mod_(*T*) can be described using Wien’s approximation of Planck’s law. The best fit to the modelled data is the function 
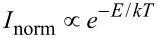
 with the energy *E* = 1.5 eV (830 nm) corresponding to the maximum spectral sensitivity of our detector. Alternatively, the often considered power-law fit was tested, which requires assuming a temperature offset *T*_0_. However even the closest fit with *T*_0_ = 850 K does not match the data well and can be used only as an approximation for higher temperatures.

**Figure 3:**
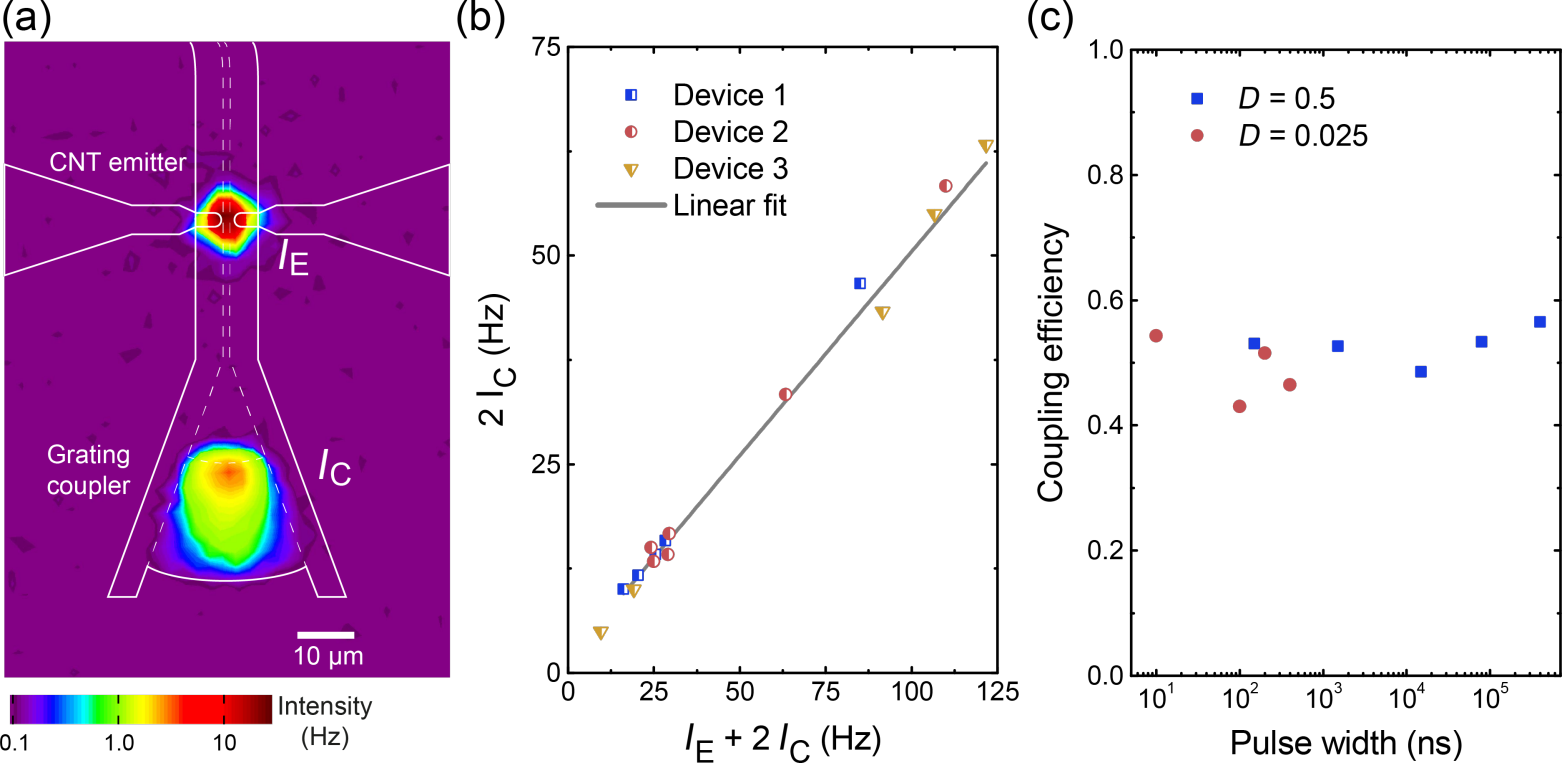
(a) Spatially resolved light emission showing intensity at the position of the CNT emitter and the grating coupler. The device geometry is indicated with white lines. The dashed lines mark the waveguide. (b) Integrated intensity recorded at the grating couplers *2I*_C_ over the sum of intensities measured at the CNT emitter and grating couplers *I*_E_ + *2I*_C_. The data were collected for three devices (blue, red and yellow symbols) in the free-space setup. A linear fit to the data provides an average coupling efficiency η ≈ 0.48. (c) Coupling efficiency for pulsed emitters with duty cycles of 0.5 (blue symbols) and 0.025 (red symbols).

Before discussing the generation of short light pulses in waveguides we would like to comment on the efficiency of the coupling of evanescent light from the CNT emitter into the waveguide. To determine the coupling efficiency we compare the emission recorded above the emitter *I*_E_ with the emission recorded above the Bragg grating coupler *I*_C_. The grating coupler serves to couple out light from the waveguide into free space. A spatially resolved emission measured on a device with a short waveguide is shown in Figure 3a.

The coupling efficiency of pulsed CNT emitters can then be calculated by η ≥ 2*I*_C_/(*I*_E_ + 2*I*_C_). Since we cannot account for loss of light due to interaction with the substrate or emission under shallow angle, we are actually considering an apparent coupling efficiency. The data measured with three devices in the broad intensity range provide an average value of η ≈ 0.48 (Figure 3b). The value of η varies in the range between 45% and 55% independent of the emission intensity and pulse width, as shown in Figure 3c. It is also comparable to previous results with DC-biased WG-CNTs [4] and exceeds the calculated values for electroluminescent CNT emitters on a SOI-waveguide by a factor of 1.5 to 2 [21].

### Optical pulses from waveguide-coupled CNTs

Finally we discuss light-pulse propagation in the waveguide studied with a fiber-coupled system. In contrast to the free-space setup, the fiber-coupled setup provides no spectral resolution, but allows us to resolve pulses with sub-nanosecond resolution in the time domain.

**Figure 4:**
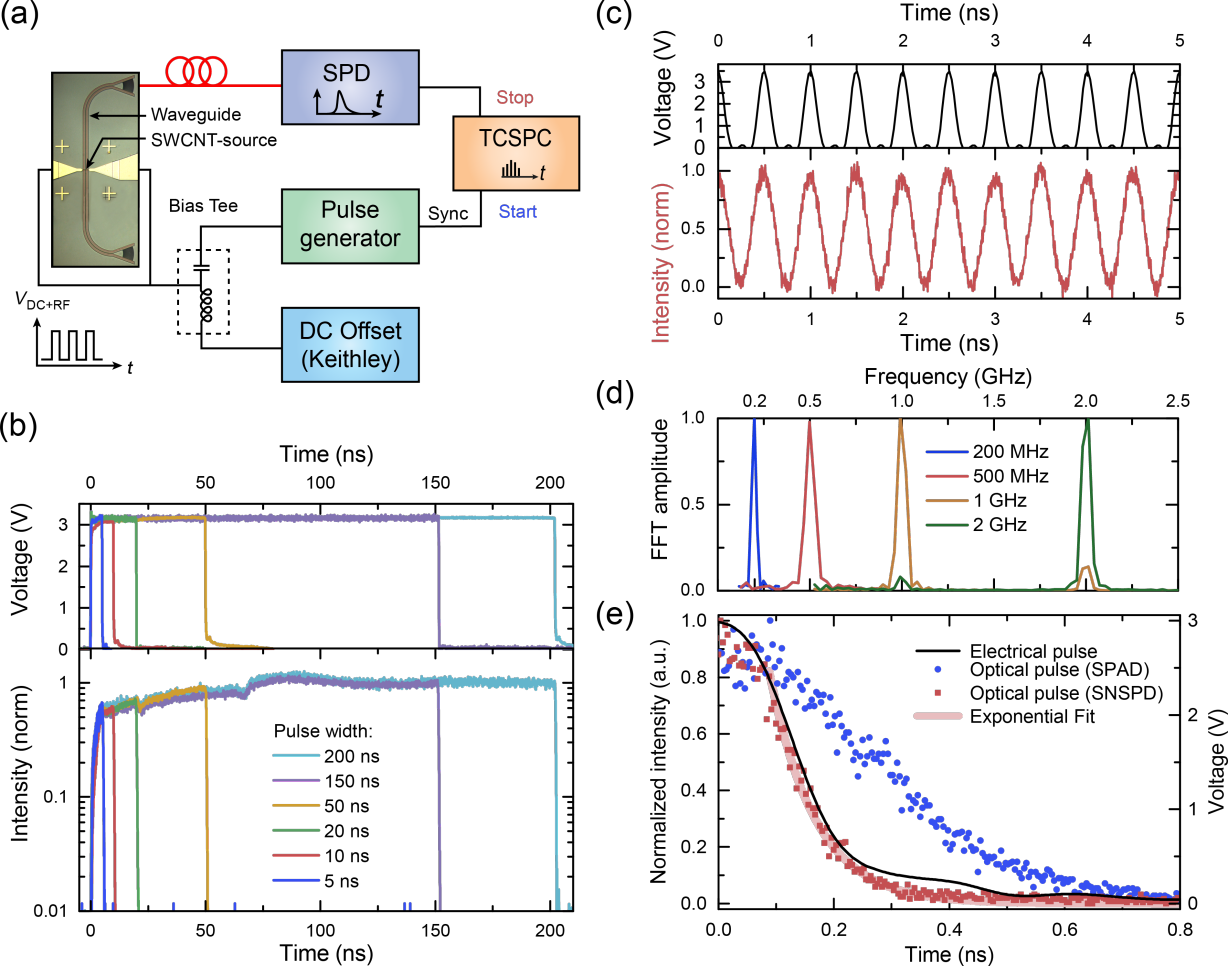
(a) WG-CNT transducer characterized by the fiber-coupled setup. (b) Comparison of electrical pulses with TCSPC-histograms of optical pulses, measured at a grating coupler. (c) Sequence of electrical pulses (150 ps width, 2 GHz, 10 V_DC_ and 3.3 V_pulse_) as well as emission pulses. (d) Normalized fast Fourier transform (FFT) spectra of modulated CNTs, emitting at frequencies of 0.2, 0.5, 1 and 2 GHz. (e) The decay of the CNT-emission following the trailing edge of an electrical pulse (black line) was measured with the slow SPAD (blue symbols) and fast SNSPD (red symbols) along with fitted exponential decay curve (decay time τ = 79 ps). The broadening of the electrical signal along with a small bump at 0.4 ns occurs because of impedance mismatch.

The operation principle is shown in Figure 4a. The CNTs were driven with electrical pulses applied with a pulse generator to the source and drain contacts via an RF-probe (Cascade Microtech). Waveguided light pulses were partly coupled out via Bragg grating couplers, designed with a central wavelength 750 ± 20 nm for guiding light into an optical fiber. Optical pulses were measured with either a single-photon avalanche detector (τ-SPAD-100, PicoQuant) or a superconducting nanowire single-photon detector [22] (SNSPD or SSPD, SCONTEL). The photon arrival times were accumulated into a histogram with 4 ps bin size using a time-correlated single-photon counting (TCSPC) unit [23] (Picoharp 300, PicoQuant), triggered by the time-synchronized pulse generator. This allows for the measurement of time-resolved, low-intensity signals and an estimation of the emission decay. The light collection efficiency of the fiber-coupled system is several orders of magnitude lower than that of the free-space setup. This is due to the limited bandwidth of the grating coupler (ca. 50 nm) and the lower numerical aperture of the fiber (NA = 0.14) compared to the microscope objective (NA = 0.42). To partially compensate the lower detection efficiency, we operated the devices at higher voltages under vacuum conditions (10^−5^ to 10^−7^ mbar). The integration time was typically set to 1–30 minutes for maximizing the signal to noise ratio. The acquired histogram was averaged over many cycles. The count rate under RF pulses was typically 1 kHz to 1 MHz.

Figure 4b shows the time-resolved optical emission versus the electrical signal amplitude applied with the pulse generator. The optical signal of the WG-CNT transducers follows the electrical signal for a pulse width ranging from 5 to 200 ns, albeit deviations are clearly visible. The dips at the relative times of 25 and 75 ns, after the electrical bias was switched on, might originate from the impedance mismatch between the pulse generator output and the RF probe along with on-chip electrical wiring. Upon switching the electrical signal on and off, we observe a fast initial response on a sub-nanosecond timescale, followed by an additional slower response on the scale of 10–100 ns. A similar behavior has been observed with electrically biased SWCNT films and was attributed to fast heating of SWCNTs and slow heating of the substrate [11]. We believe that this interpretation holds also for the response of our WG-CNT transducers. However, for shorter pulses the slow heating does not set in before the end of the pulse, as shown in Figure 4c. The data demonstrates a sequence of 150 ps wide pulses at 2 GHz repetition rate, which constitutes the fastest modulation for CNT transducers so far. The Fourier transformations of TSCPC histograms demonstrate the modulation of photon emission measured with different devices at 200 MHz, 500 MHz, 1 GHz and 2 GHz (Figure 4d). Higher repetition rates are limited by the setup.

In theory, the characteristic timescale of a thermal emitter τ_therm_ solely depends on the mass density ρ_CNT_, the specific heat capacitance *c*_CNT_, and thermal conductance *g* between the CNTs and the substrate, as pointed out previously [11,19]: τ_therm_ = ρ_CNT_·*c*_CNT_/*g*. For our CNTs with a diameter of 0.8–1.2 nm at 1000–1500 K, ρ_CNT_ varies between 0.7·10^−15^ and 1.8·10^−15^ kg/m, *g* is in the range of 0.1–0.3 W/K·m [19] and *c*_CNT_ = 2500–3900 Ws/kg·K [24]. *c*_CNT_ and consequently *τ*_therm_ increases with CNT diameter and with temperature. The calculated *τ*_therm_ for the parameter range given above is 5–70 ps, making CNTs an ultra-fast thermal light source.

Indeed by using an ultra-fast single-photon detector, we were able to prove that our WG-CNT transducers have a characteristic time scale for signal conversion of about 80 ps. Figure 4e shows measurements using single-photon detectors with different time resolutions given by the timing jitter of the detectors (350 ps for τ-SPAD and 40 ps for SNSPD for a wavelength range of 700–900 nm).

Upon switching off the electrical signal, the emission intensity decays exponentially. Experimentally, τ_therm_ extracted from a time-domain histogram (Figure 4e, measured with SNSPD) is somewhat larger than the theoretically expected value, which could be due to the decay time of electrical pulses τ_pulse_ ≈ 80 ps, as measured on chip (Figure 4e, black line). Moreover, the cumulative timing jitter of detector, pulse generator and connectors lead to additional broadening of the light pulse and thus increase of the measured decay time. Due to these instrumental restrictions, we are not able to determine the upper limit of the switching rate for the presented waveguide-coupled CNT-based light emitter. However, even the demonstrated rates are exceptionally high for an integrated thermal emitter on chip. Both dense films as well as single CNTs emerge as reproducible, stable light sources in the gigahertz range within our experimental resolution.
